# Association between Radiotherapy and Risk of Cancer Associated Venous Thromboembolism: A Sub-Analysis of the COMPASS—CAT Study

**DOI:** 10.3390/cancers13051033

**Published:** 2021-03-02

**Authors:** Sally Temraz, Nour Moukalled, Grigorios T. Gerotziafas, Ismail Elalamy, Luis Jara-Palomares, Maya Charafeddine, Ali Taher

**Affiliations:** 1 Department of Internal Medicine, Division of Hematology/Oncology, American University of Beirut, Beirut 1107 2020, Lebanon; st29@aub.edu.lb (S.T.); nour.mmoukalled@gmail.com (N.M.); mc16@aub.edu.lb (M.C.); 2 Cancer Biology and Therapeutics, INSERM UMR S938, Institut Universitaire de Cancérologie (IUC), Sorbonne Université, 75012 Paris, France; gregtnn@gmail.com (G.T.G.); ismail.elalamy@aphp.fr (I.E.); 3 Haemostasis and Thrombosis Centre, Biological Hematology Department, Hôpital Tenon, AP-HP Sorbonne Université, CEDEX 20, 75970 Paris, France; 4 Department of Obstetrics and Gynecology, I.M. Sechenov First Moscow State Medical University, 119991 Moscow, Russia; 5 Respiratory Department, Medical Surgical Unit of Respiratory Diseases, Hospital Virgen del RocIo, 41013 Seville, Spain; luisoneumo@hotmail.com; 6 Centro de Investigación Biomédica en Red de Enfermedades Respiratorias (CIBERES), Instituto de Salud Carlos III, 28029 Madrid, Spain

**Keywords:** neoplasms, pulmonary embolism, risk factors, radiotherapy, venous thromboembolism

## Abstract

**Simple Summary:**

Cancer patients are at an increased risk of developing venous thromboembolism (VTE) compared to non-cancer patients. VTE in cancer patients poses as a financial burden and influences quality of life and is correlated with increased morbidity and mortality. Several cancer-related and patient-related risk factors have been shown to be predictors of VTE in cancer patients. However, the effect of radiotherapy on development of thrombosis in cancer patients is not extensively explored. In this report, radiotherapy was significantly associated with increased risk for VTE. The risk of VTE was higher in women, patients >50 and those receiving chemo- or hormonal therapy.

**Abstract:**

Background: The role and effect of radiotherapy in the development of VTE has not been extensively explored; Methods: This is a post-hoc analysis from the COMPASS-CAT trial. Patients with breast, lung, colon or ovarian cancer, with early, locally advanced or metastatic disease and receiving chemotherapy were included. Primary endpoint was documented symptomatic VTE; Results: A total of 1355 patients were enrolled between November 2013 and November 2015. Of those, 194 patients were excluded because of missing data or the use of anticoagulation. Of the evaluable patients, 361 patients received radiotherapy (33.6%) At a median follow up of 6 months, 9.1% (n = 33) of patients receiving radiotherapy developed a VTE event (excluding those with missing data on follow up). After applying the competing risk model, radiotherapy remained significantly associated with increased risk for VTE (HR 2.47, 95% CI: 1.47–4.12, *p* = 0.001). Stratification analysis for the cohort that received radiotherapy revealed an increased risk of VTE in women compared to men (10.8% vs. 2.7%; *p* = 0.03), in those older than 50 (12.2% vs. 3.7%; *p* = 0.011); for patients receiving anthracycline chemotherapy (14.4% vs. 2.9%; *p* < 0.001) and hormonal therapy (12.9% vs. 3.9%; *p* < 0.001); Conclusions: Analysis from the COMPASS-CAT revealed a significant correlation between radiotherapy and VTE in patients with cancer. Further studies are needed to better understand the potential cellular toxicity associated with radiotherapy.

## 1. Introduction

Cancer patients have a four- to six-fold increased risk of developing venous thromboembolism (VTE) compared to non-cancer patients, with an incidence approaching up to 19% depending on the type and stage of the tumor [1,2,3]. VTE has been related to significant consequences, where post-operative thromboembolic events were associated with an average excess charge of $USD 21,709 per hospital stay [4]. In addition to the economic burden, the development of VTE in cancer patients influences their quality of life and entails significant morbidity and mortality [5,6]. VTE is the second most common cause of death among hospitalized cancer patients [7]. Danish mortality data revealed that cancer patients who develop thromboembolic events have a significantly lower one year survival compared to those without thrombosis (12% versus 38%, respectively) [8]. Guidelines have been formulated to improve the perception of thrombotic risk in cancer patients and ensure the adoption of appropriate thromboprophylaxis. Clinical trials have identified specific iatrogenic factors that would further increase the risk of thrombotic events, including the presence of indwelling venous catheters, prior surgeries, chemotherapeutic and hormonal agents [9,10,11]. Nonetheless, VTE remains an under-recognized complication of cancer and cancer treatment modalities. Much is yet to be unraveled regarding the effect of various chemotherapeutic; immunotherapeutic; as well as targeted agents on the thrombotic risk.

Radiotherapy (RT) has recently become a cornerstone in the management of various types of malignancies, whether for curative or palliative indications. More than a third of patients diagnosed with any type of cancer will be treated with radiotherapy during the course of their disease, either in the early period post diagnosis where the risk of VTE has been found to be high [12,13], or later during advanced stages of the disease when the functionality declines and the cumulative risk for thrombosis increases significantly. RT is the standard of care with concurrent chemotherapy for stage III inoperable lung cancer, in the adjuvant setting after breast conserving surgeries for breast cancer, or after resection of lung tumors with evidence of mediastinal lymphadenopathy, in the treatment of head and neck cancers; as well as, for specific types of gastro-intestinal and genito-urinary malignancies. However, its association with thromboembolic events has been less evaluated.

The multicenter, prospective, longitudinal, non-interventional Prospective Comparison of Methods for thromboembolic risk assessment with clinical Perceptions and AwareneSS in real life patients-Cancer Associated Thrombosis (COMPASS–CAT study) was performed to identify significant risk factors for the development of VTE in patients with four types of solid tumors frequent in the community who receive chemotherapy as outpatients. This study led to the derivation of a new risk assessment model (RAM) for CAT in outpatients with one of the abovementioned types of cancer. The major predictors for VTE risk included in COMPASS-CAT RAM include cancer-related risk factors such as anti-hormonal and anthracycline treatment, stage and time since cancer diagnosis and central venous catheter use, as well as patient related risk predictors such as cardiovascular risk factors, recent hospitalization as well as personal history of VTE and platelet counts [12]. We have herein analyzed the effect of RT on this risk, which was not incorporated in the COMPASS-CAT RAM model. In this post-hoc analysis from the COMPASS-CAT trial, we aimed to evaluate the association of RT with VTE in cancer patients.

## 2. Results

**Figure 1:**
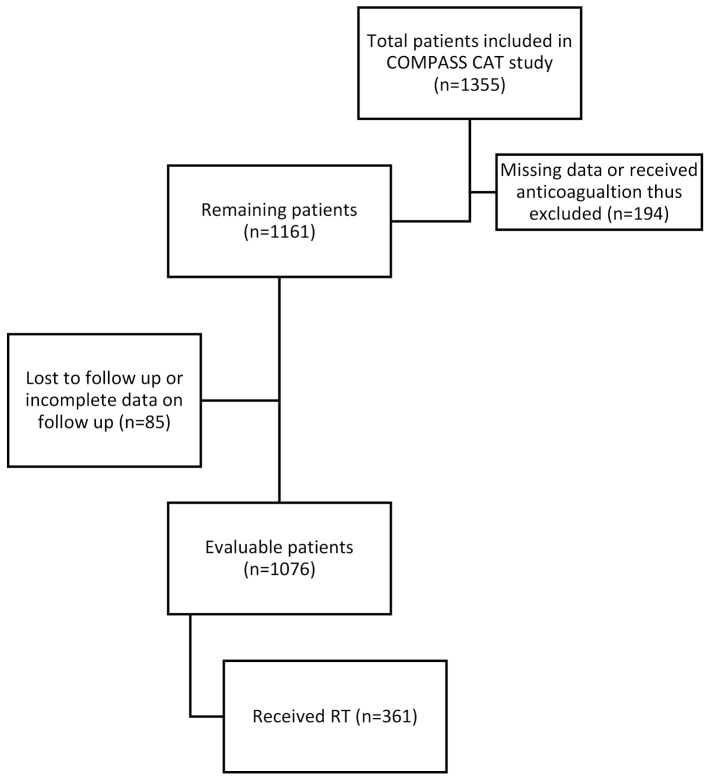
Flow chart of patients from COMPASS-CAT study included in the competing-risks regression model.

A total of 1355 patients were enrolled. Of those, 194 were ultimately excluded for missing data or because of receiving anticoagulation after enrollment, while 85 patients were either lost to follow up or had incomplete data on follow up (Figure 1). Table 1 shows the patient characteristics of the evaluable patients at 6 months, and those who received RT among this cohort. The analysis included 59.3% breast cancer patients (n = 638), 19.5% colorectal cancer patients (n = 210), 12.6% lung cancer patients (n = 136) and 8.6% ovarian cancer patients (n = 92). At enrollment, around 37.7% of evaluable patients (n = 405) had metastatic disease. The one-year mortality rate in the whole cohort was 9.4%.

Radiotherapy (RT) was administered in 33.6% of evaluable patients (n = 361). The median age in the RT group was 54 years. This group included 79.5% women (n = 287), and 288 patients with an Eastern Cooperative Oncology Group (ECOG) ≤1 (valid percentage 84%). The main anti-cancer treatment administered in patients who received RT was anthracycline therapy in 194 patients (valid percentage 58.6%). Only 6.6% of those patients (n = 24) had a personal history of DVT.

At a median follow up of 6 months, 9.1% (n = 336) of those that received RT developed a VTE event (excluding those with incomplete data on follow up). VTE events included 28 distal lower extremity deep venous thrombosis, two pulmonary embolisms, one upper extremity thrombosis, one central venous catheter related thrombosis and one other location.

We used the competing risk model to evaluate the risk of thrombosis associated with RT given the high disease-specific mortality. Of the total VTE cases, 16 were excluded because the date of the event was not available. Table 2 shows the steps taken in the competing risk model using the backward conditional model. After applying this model, radiotherapy remained significantly associated with increased risk for VTE (HR 2.47, 95% CI: 1.47–4.12, *p* = 0.001). Older age increased VTE risk in the first step but the difference was not significant. Patients who received anthracycline containing regimens surprisingly had a lower risk of VTE, nearly as half of their counterparts (HR 0.55, 95% CI: 0.31–0.98, *p* = 0.045) as per this competing risk model. As for all the remaining variables, these were not associated with a significantly increased risk of VTE (Table 2).

Table 3 presents the Fischer exact test for VTE in the radiotherapy cohort across various categories of patients. This stratification revealed that there is a statistically significant increased risk of VTE in women compared to men (10.8% vs. 2.7%; *p* = 0.03), in those older than 50 years of age compared to younger patients (12.2% vs. 3.7%; *p* = 0.011); as well for patients who received anthracycline chemotherapy (14.4% vs. 2.9%; *p* <0.001) and/or hormonal therapy (12.9% vs. 3.9%; *p* <0.001). When comparing between cancer types, breast cancer was significantly associated with a higher risk for VTE (11% vs. 3%; *p* = 0.042), colorectal cancer was associated with a lower risk for VTE but was also statistically significant (2.8% vs. 16.4; *p* = 0.038).

## 3. Discussion

The present post-hoc analysis of the prospective multicenter COMPASS-CAT trial shows that radiotherapy is an independent risk factor for CAT in patients with breast, lung, ovarian or colon cancer treated with chemotherapy. The strengths of this analysis lie in the multicenter international nature of the study that encompasses a large number of patients, and the fact that it was performed in the outpatient setting; in addition to the long duration of follow up. Our results are further confirmed by other small or retrospective studies. Until recently, only small case series have suggested a higher risk of thromboembolic events associated with RT [14]. However, with the growing knowledge regarding the morbidity/mortality associated with VTE, investigators have become interested in the effect of RT on VTE risk; in addition to the outcome of cancer patients developing VTE events in this setting. A retrospective analysis of outpatient cancer patients, who were treated with RT or chemotherapy, was conducted to assess the impact of RT on the risk of CAT. In this study, patients were divided into three groups including 165 treated with 3-Dimensional conformal RT for brain tumors or brain metastasis (10 patients-around 6% had VTE), 158 treated with RT for body tumors (four patients had VTE), and a third control group including 164 patients treated with chemotherapy (four patients had VTE) [15]. External beam RT was associated with a significantly increased risk of VTE compared to chemotherapy, with a risk difference of 5% (*p* = 0.018). In our post-hoc analysis of the COMPASS-CAT study, RT was associated with an increased risk of VTE in cancer patients on multivariate analysis (*p* = 0.001). In the RT group, we documented a higher risk for VTE among women compared to men (taking into account that around 80% of the radiotherapy cohort were women); in patients older than 50 years of age, in addition to those who received anthracycline-based chemotherapy and/or hormonal therapy, suggesting that, although this data need to be confirmed, these factors need to be considered when evaluating the risk for VTE in patients treated with radiotherapy. Women are at an increased risk of VTE mostly due to the use of hormone therapy and pregnancy [16]. Use of chemotherapy reduces protein C and protein S levels, and exerts detrimental effect on endothelial cells, all of which contribute to the pathogenesis of VTE [17,18]. As for hormone therapy, specifically tamoxifen, the increased risk can be explained by the altered circulating coagulation inhibitors induced by tamoxifen, including reduced antithrombin, protein C levels, and protein S levels [17,18].

We acknowledge that this sub-analysis shows different results in terms of the significant risk factors for development of VTE compared to the previously published data used for creation of the VTE risk assessment model. However, as previously reported, the multivariable logistic regression model was used to explore the effect of independent variables on VTE risk, in order to create a risk assessment model for stratification of patients regarding the risk for VTE. This model identifies the variables that affect the outcome in a dichotomous way only. For this evaluation, only patients who were evaluable at 6 months were included in the derivation cohort. On the other hand, the current sub-analysis, was mainly intended to study the significance of radiotherapy as a risk factor for VTE, to evaluate the need for further trials to confirm this association, thus given the death rate in this cohort, the competing risk analysis was utilized in this evaluation, and this accounts for both the presence of the risk factor as well as the time-to-event. In addition, the competing risk analysis eliminates the bias associated with death. Using this competing risk model, anthracycline chemotherapy was associated with a lower risk for VTE, as opposed to what had been previously published in the risk assessment model. We believe that this could be partly explained by the fact that the majority of our patients had breast cancer which is commonly treated with anthracyclines, and which is characterized by a longer median overall survival compared to the other included solid tumors. Thus, this protective effect could be related to the type of cancer rather than type of treatment. We believe that we cannot draw specific conclusions regarding the effect on anthracyclines in combination with radiotherapy at this point.

Other data on VTE in RT comes from disease specific trials investigating the impact of RT in certain gastro-intestinal and/or genitourinary cancers. Analyzing data for around 9000 Swedish male patients treated with curative radiotherapy for prostate cancer, Bosco et al. reported a positive correlation between external beam radiotherapy and brachytherapy and the risk of PE, which later became statistically insignificant after adjusting for confounders, indicating less alterations induced by RT on large pelvic veins [19]. Furthermore, the Stockholm I and II trials indicated a higher risk of VTE among irradiated patients with rectal cancer [20], where reports from the latter study indicate that cardiovascular complications were the most important cause of early post-operative deaths in these patients [21]. On the other hand, mixed results have been reported in various studies evaluating the cardiovascular complications, including VTE, in association with RT in rectal cancer patients. Nonetheless, pooled data from a large population-based registry and 4 randomized clinical trials of patients with rectal cancer treated with pre-operative radiotherapy or chemo-radiotherapy versus surgery alone, revealed a higher risk of DVT in contrast to other cardiovascular events, mainly during the first 6 months after surgery, but with a small absolute number of patients [22]. Our stratification analysis revealed a statistically significant increase in the risk for VTE in breast cancer compared to other malignancies; however, these findings might be affected by the proportion difference across the four types of cancer included. Colorectal cancer was associated with a lower risk for VTE compared with the others (statistically significant), and this needs further exploration in future trials examining the effect of RT on VTE.

Multiple proposed mechanisms might explain the increased risk of thrombosis during and after radiotherapy. Radiation can enhance secondary venous hemostasis, stimulate release of inflammatory molecules [23], which leads to activation of the endothelium and creates a prothrombotic environment [24]. In addition, ionizing radiation has been shown to affect multiple prothrombotic molecules (activated factor VIII, NF-kappa B, D-dimers, platelet, tissue factor and von Willebrand activation) [25,26], and anticoagulant molecules (thrombomodulin and protein C) [27], thus tipping the balance towards a hypercoagulable state.

The optimal therapy with regards to dose and duration of thromboprophylaxis in cancer patients receiving RT is still lacking. Thus, anticoagulation therapy is still non-standardized and relatively variable. Heparin appears to have no effect on mortality at 12 months and 24 months [28]. It reduces symptomatic VTE; however, increases the likelihood of major and minor bleeding. Reports from a recent living systemic review that included randomized controlled trials assessing the benefits and harms of vitamin K antagonists or direct oral anticoagulants in ambulatory patients with cancer did not show a mortality benefit from oral anticoagulation, but suggested an increased risk for bleeding [29]. Also, the RIETE registry showed that patients treated with radiotherapy are two times more likely to develop cerebral bleeding once started on therapeutic anticoagulation [30], which further strengthens the need to properly identify high risk groups, and the possible role of thromboprophylaxis to prevent both thrombotic and bleeding complications.

Limitations of this analysis include the variability of patients, related to the multinational nature of the study with patients included across six different countries which are characterized by variable additional risk factors for VTE (lifestyle and genetic disorders among others) with a difficulty to account for this heterogeneity despite including the country in our analysis, the lack of data regarding the delivered radiotherapy doses, volumes; as well as, the interval between radiotherapy exposure and the development of VTE, which need to be addressed in future trials. The limitations of the present study also relate to the various tumor types that were studied. The radiotherapy regimens used for these tumor types vary widely by cumulative dose and site of radiotherapy, and even within tumor types there is heterogeneity. Hence, it is difficult to translate the findings to clinical practice. For example, the mechanism maybe different in ovarian cancer, where direct endothelial damage could lead to DVT, compared to lung cancer where it may be secondary prothrombotic effects. Another limitation involves the study results being driven by the large number of breast cancer patients where most of the VTE events occurred.

## 4. Materials and Methods

The COMPASS-CAT study was an investigator-initiated multinational prospective observational trial. Consecutive patients with histologically confirmed breast, lung, colon or ovarian cancer, whether early stage, locally advanced or metastatic disease, who were receiving chemotherapy in the outpatient department at six institutions in France, Lebanon, Jordan, Saudi Arabia, Kuwait and Syria, were enrolled and followed up for 12 months. Exclusion criteria included age younger than 18 years, life expectancy of less than 3 months, pregnancy or psychiatric disorders, a documented VTE episode or acute coronary syndrome during the past 6 months, recent hospitalization for cardiovascular diseases or respiratory failure, ongoing anticoagulation therapy, or planned curative surgery. Patients were recruited from November 2013 to November 2015. The primary endpoint was objectively documented symptomatic thromboembolic events including DVT, pulmonary embolism (PE), central venous catheter thrombosis, and/or thrombosis at rare venous sites [12].

Patients were followed up at 3, 6 and 12 months for the development of VTE events, bleeding episodes, disease status, and supportive treatments used. The confirmation of thrombotic events was done by Echo-Doppler, computerized tomography, magnetic resonance imaging, angiography, or scintigraphy. Patients that developed VTE but are missing the date that VTE developed (n = 16) were excluded because no time-to-event can be computed for them. The study was approved by the institutional review boards or ethics committees of participating institutions, and all enrolled patients provided written informed consent. The study was conducted in compliance with the recognized international standards, including the International Conference On Harmonization (ICH) and the principles of the Declaration of Helsinki.

Descriptive statistics included frequencies of the categorical variables, while mean, median and range were summarized for numerical variables. Cross-tabulations in the form of 2 × 2 tables were plotted to compare and predict variables for the VTE event, Fisher’s exact test was considered to evaluate significance instead of Pearson’s chi square in stratifications with low counts. The multivariable analysis was done based on previous assessment done by the authors, including in this analysis RT. In order to understand the role of variables influencing VTE event at 6 months, the new risk assessment model included the following risk factors: radiotherapy, age, body mass index BMI, personal/family history of VTE, hormonal therapy, chemotherapy, antiangiogenic therapy surgery, cvc, hospitalization, hormonal therapy, anthracycline containing therapy, platelet count  ≥350 G/L, comorbidities, cardiovascular risk factors, time since cancer diagnosis ≤6 months, and advanced stage of cancer. In a patient population with high mortality, disease-related deaths could affect the risk of death from other causes referred to as a competing event. To alleviate this bias, the competing risk model was applied, given that it censors observations that had a competing death event instead of the event of interest, as opposed to the Cox regression analysis.

The competing risk analysis accounts for death as a modifier in the chance of having a VTE event, thus better estimating the probability for cause-specific events than traditional survival analyses. The competing event is defined initially as patients who passed away in the first three months following their first visit, as such, these observations are considered as belonging to a competing risk. The next step and interpretation are similar to a Cox-regression analysis in which a time-to event is calculated from the visit date to the date of event of interest in months, and all independent variables are added to the model. In order to assess the role of each of the independent variables on the event of interest, the first step consisted of adding all variables at once, the hazard ratios and their corresponding 95% confidence intervals are calculated. Each hazard ratio is calculated controlling for other variables in the model. In order to retain the most significant independent variables, the analysis was done using backward conditional model in which all variables are entered in the equation at first and then each of the variables is sequentially removed at each step when considered insignificant to the model following a criterion of *p* > 0.1. The final model retains the significant variables, the cause-specific hazard ratios (ORs) and 95% CIs were calculated to estimate their risk on the event of interest. A value of *p* < 0.05 was considered significant in all analyses. All statistical analysis was performed using the SPSS v.25.0 statistical package (IBM Corp, Armonk, NY, USA) and STATA v.14.0 (STAT corp., College Station, TX, USA).

## 5. Conclusions

Analysis from the COMPASS-CAT revealed a significant correlation between radiotherapy and the risk of VTE in patients with cancer. The development of thromboembolic events in cancer patients is associated with significant morbidity and mortality. Further studies are thus needed to better understand the potential cellular toxicity associated with radiotherapy.

## Figures and Tables

**Table 1:** Baseline characteristics for evaluable patients and those who received radiotherapy *.

Characteristics	Total Evaluable Patients (n = 1076)	Patients who Received RT of Evaluable Patients (n = 361)
Age (years)	54	54
Women, n (%)	868 (80.7%)	287 (79.5%)
BMI > 30, n (%)	258 (23.9%)	75 (21.2%)
Comorbidities, n (%)		
- Hypertension	283 (26.4%)	79 (21.9%)
- Diabetes Mellitus	123 (11.4%)	46 (12.7%)
- CAD	52 (4.8%)	26 (7.2%)
- Renal impairment	26 (2.4%)	5 (1.4%)
- Liver impairment	34 (3.2%)	2 (0.6%)
- Heart failure NYHA class I/II	17 (1.6%)	5 (1.4%)
- Heart failure NYHA class III/IV	2 (0.2%)	1 (0.3%)
- Peripheral vascular disease	12 (1.1%)	6 (1.7%)
- Personal history of VTE	68 (6.4%)	24 (6.6%)
- Family history of VTE	74 (7%)	22 (6.1%)
Type of cancer, n (%)		
- Breast	638 (59.3%)	264 (73.1%)
- Colon	210 (19.5%)	72 (20%)
- Lung	136 (12.6%)	22 (6.1%)
- Ovarian	92 (8.6%)	3 (0.8%)
Stage of cancer, n (%)		
- Localized	356 (33.2%)	135 (37.7%)
- Locally advanced	312 (29.1%)	132 (36.9%)
- Metastatic	405 (37.7%)	91 (25.4%)
Time since diagnosis, n (%)		
- ≤ 6 months	653 (60.7)	252 (69.8%)
ECOG Performance, n (%)		
- ≤1	816 (91.6%)	288 (84%)
Type of anticancer therapy, n (%)		
- Anthracycline containing	402 (42.7%)	194 (58.6%)
- Platinum chemotherapy	261 (24.3%)	31 (9.4%)
- Antiangiogenic therapy	210 (19.5%)	41 (11.4%)
- Anti-hormonal therapy	302 (28.1%)	209 (57.9%)

* Percentages were calculated excluding missing entries from the denominator in each variable (valid %).

**Table 2:** Competing-risks regression model examining variables influencing VTE at 6 months *.

Variable	Coefficient	95% CI	*p*-Value
Step 1: regression model includes all variables in the model *p* = 0.0037			
Chemotherapy	0.87	0.36–2.15	0.773
Family history of VTE	1.07	0.42–2.70	0.892
Antiangiogenic agent	0.95	0.47–1.93	0.894
Radiotherapy	2.34	1.41–3.90	<0.001
BMI	1.00	0.98–1.04	0.610
Age	1.02	1.00–1.05	0.067
Surgery	1.59	0.71–3.57	0.262
CVC	1.07	0.59–1.95	0.823
Hormonal therapy	1.73	0.91–3.28	0.093
Anthracycline containing therapy	0.44	0.23–0.84	0.013
Platelet count ≥ 350 G/L	0.87	0.50–1.50	0.611
Comorbidities	1.29	0.67–2.53	0.456
Cardiovascular risk factors	0.64	0.33–1.24	0.189
time since cancer diagnosis ≤6 months	1.19	0.65–2.20	0.566
advanced stage of cancer	0.91	0.52–1.62	0.771
Final step: regression model includes significant variables only			
Radiotherapy	2.47	1.48–4.13	0.001
Age	1.02	1.00–1.04	0.064
Anthracycline containing therapy	0.55	0.31–0.99	0.045

Variables entered in the model: chemotherapy, radiotherapy, family history of VTE, antiagiogenic agent, BMI, age, surgery, cvc, hormonal therapy, anthracycline containing therapy, platelet count ≥350 G/L, comorbidities, cardiovascular risk factors, hospitalization, time since cancer diagnosis ≤6 months, advanced stage of cancer. * Backward step conditional binary logistic analysis in which all variables are entered in the equation and then sequentially removed at each step when deemed insignificant to the model.

**Table 3:** Fischer exact test for VTE in the radiotherapy cohort across different categories *.

Variable	VTE (%) (n = 33)	*p*-Value
Gender		0.03
- Men (n = 74)	2 (2.7%)	
- Women (n = 287)	31 (10.8%)	
Age categories		0.011
- ≤50 (n = 108)	4 (3.7%)	
- >50 (n = 237)	29 (12.2%)	
Type of Cancer		
- Breast (n = 64)	29 (11%)	0.042
- Colorectal (n = 72)	2 (2.8%)	0.038
- Lung (n = 22)	2 (9%)	1
- Ovarian (n = 3)	None	NA
Treatments		
- Anthracyclines (n = 194)	28 (14.4%)	0.001
- Platinum containing (n = 31)	1 (3.0%)	0.753
- Antiangiogenic (n = 41)	2 (4.9%)	0.40
- Hormonal therapy (n = 209)	27 (12.9%)	0.005
- Surgery (n = 314)	30 (9.6%)	0.472

* Percentages were calculated excluding missing entries from the denominator in each variable.

## Data Availability

The data presented in this study are available on request from the corresponding author. The data are not publicly available due to ethical considerations.
